# The relationship among the progression of inflammation in umbilical cord, fetal inflammatory response, early-onset neonatal sepsis, and chorioamnionitis

**DOI:** 10.1371/journal.pone.0225328

**Published:** 2019-11-19

**Authors:** Jeong-Won Oh, Chan-Wook Park, Kyung Chul Moon, Joong Shin Park, Jong Kwan Jun

**Affiliations:** 1 Department of Obstetrics and Gynecology, Division of Maternal Fetal Medicine, Seoul National University College of Medicine, Seoul, Korea; 2 Department of Pathology, Seoul National University College of Medicine, Seoul, Korea; University of Wisconsin - Madison, School of Veterinary Medicine, UNITED STATES

## Abstract

**Objectives:**

No information exists about whether fetal inflammatory-response(FIR), early-onset neonatal sepsis(EONS) and chorioamnionitis(an advanced-stage of maternal inflammatory-response in extraplacental membranes) continuously increase according to the progression of inflammation in umbilical-cord(UC). The objective of current-study is to examine this-issue.

**Methods:**

Study-population included 239singleton pregnant-women(gestational-age[GA] at delivery: 21.6~36weeks) who had inflammation in extraplacental membranes or chorionic plate (CP) and either preterm-labor or preterm-PROM. We examined FIR, and the frequency of fetal inflammatory-responses syndrome(FIRS), proven-EONS, suspected-EONS and chorioamnionitis according to the progression of inflammation in UC. The progression of inflammation in UC was divided with a slight-modification from previously reported-criteria as follows: stage0, inflammation-free UC; stage-1: umbilical phlebitis only; stage-2: involvement of at least one UA and either the other UA or UV without extension into WJ; stage-3: the extension of inflammation into WJ. FIR was gauged by umbilical-cord-plasma(UCP) CRP concentration(ng/ml) at birth, and FIRS was defined as an elevated UCP CRP concentration at birth(≥200ng/ml).

**Results:**

Stage-0, stage-1, stage-2 and stage-3 of inflammation in UC were present in 48.1%, 15.5%, 6.7%, and 29.7% of cases. FIR continuously increased according to the progression of inflammation in UC(Kruskal-Wallis test,P<0.001; Spearman-rank-correlation test,P<0.000001,r = 0.546). Moreover, there was a significant and stepwise increase in the frequency of FIRS, proven-EONS, suspected-EONS and chorioamnionitis according to the progression of inflammation in UC(each for P<0.000005 in both chi-square test and linear-by-linear-association). Multiple logistic-regression analysis demonstrated that the more advanced-stage in the progression of inflammation in UC(i.e., stage-1 vs. stage-2 vs. stage-3), the better predictor of suspected-EONS (Odds-ratio[OR]3.358, 95%confidence-interval[CI]:1.020–11.057 vs. OR5.147, 95%CI:1.189–22.275 vs. OR11.040, 95%CI:4.118–29.592) and chorioamnionitis(OR6.593, 95%CI:2.717–15.999 vs. OR16.508, 95%CI:3.916–69.596 vs. OR20.167, 95%CI:8.629–47.137).

**Conclusion:**

FIR, EONS and chorioamnionitis continuously increase according to the progression of inflammation in UC among preterm-gestations with inflammation in extraplacental membranes or CP. This finding may suggest that funisitis(inflammation in UC) is both qualitatively and quantitatively histologic-counterpart of FIRS, and a surrogate-marker for chorioamnionitis.

## Introduction

Ascending intrauterine infection/inflammation (AIUI) is a central pathophysiology of spontaneous preterm birth due to either preterm labor and intact membranes (PTL) or preterm premature rupture of membranes (preterm-PROM) [1–3]. AIUI proceeds to amniotic cavity through the choriodecidua (CD) and amnion, and finally leads to the invasion of fetus [1–3]. During the progress of AIUI, acute inflammation by neutrophil infiltration develops in the CD of extraplacental membranes as an early stage of AIUI, and subsequently occurs in the amnion of extraplacental membranes and the umbilical cord (UC) of fetus as an advanced stage of AIUI, eventually resulting in fetal inflammatory response (FIR) [4–7]. Funisitis (inflammation in UC) defined in the presence of neutrophil infiltration into the umbilical vessels with or without the involvement of Wharton’s Jelly (WJ) in fetus is known to be the histologic hallmark of systemic fetal inflammation, fetal inflammatory response syndrome (FIRS) [6–8]. In general, inflammation of the umbilical vessels begins in umbilical vein (UV) (umbilical phlebitis) and is followed by involvement of either umbilical artery (UA) or umbilical arteries (UAs) (umbilical arteritis) [9], and the extension of inflammation into WJ is considered to be a more advanced stage than inflammation restricted to umbilical vessels based on the finding that the presence of extension of neutrophils into WJ was associated with a more increased FIR than the absence of that lesion in the context of inflammation in UV and/or UA [10].

Both inflammation in UC [6, 11] and FIRS [7, 8] are closely associated with fetal infectious morbidity such as early-onset neonatal sepsis (EONS) and chorioamnionitis as an advanced stage of maternal inflammatory response (MIR) in extraplacental membranes [5]. Therefore, it is plausible that either the intensity of FIR or the frequency of EONS increases according to the progression of inflammation in UC. However, there are only a few studies examining the intensity of FIR according to the progression of inflammation in UC [10, 12, 13]. Of note, they all had the limitation in either the classification of inflammation in UC [12, 13] or the number of study population [10, 13], and moreover, their findings were inconsistent between studies [10, 12, 13], thereby failing to draw a conclusion that FIR continuously increases according to the progression of inflammation in UC from umbilical phlebitis only via involvement of at least one UA to the extension of inflammation into WJ. Moreover, we could find only one report examining the relationship between the progression of inflammation in UC and EONS [14]. Unfortunately, that study reported that there was not a stepwise increase in EONS according to the progression of inflammation in UC due to the limitation in classification, and did not present the status of FIR according to the progression of inflammation in UC [14]. Furthermore, no study has ever demonstrated that chorioamnionitis more frequently developed according to the progression of inflammation in UC, although chorioamnionitis was associated with an increase in the frequency of inflammation in UC [5]. However, there is a good chance for the more frequent development of chorioamnionitis according to the progression of inflammation in UC, considering that both chorioamnionitis and inflammation in UC are an advanced stage of AIUI [1–3].

In summary, up to now, there is no information whether FIR, EONS, and chorioamnionitis continuously increase according to the progression of inflammation in UC (i.e., umbilical phlebitis only, involvement of at least one UA and either the other UA or UV without extension into WJ, and the extension of inflammation into WJ). We hypothesized that the intensity of FIR, and the frequency of FIRS, EONS and chorioamnionitis would be continuously increased with the progression of inflammation in UC (i.e., stage 0, inflammation-free UC; stage 1, umbilical phlebitis only; stage 2, involvement of at least one UA and the other UA or UV without extension into WJ; and stage 3, the extension of inflammation into WJ). The objective of the study is to examine this issue.

## Materials and methods

### Study design

The study population included of 239 singleton pregnant women who delivered at the Seoul National University Hospital, and met the following conditions: 1) gestational age (GA) at delivery: 21.6~36 weeks; 2) preterm birth due to either PTL (101 cases) or preterm-PROM (138 cases); and 3) the presence of inflammation in extraplacental membranes or CP. At our institution, UC blood was routinely obtained to all patients who were admitted with the diagnosis of either PTL or preterm-PROM. PTL was defined as the presence of regular uterine contractions with a frequency of at least two every 10 minutes and cervical changes in the context of intact membranes, and rupture of membranes (ROM) was diagnosed by examination with sterile speculum confirming pooling of amniotic fluid in the vagina, a positive nitrazine paper test results and a positive ferning test result as previously reported [15, 16]. Moreover, we routinely recommended and performed placental pathologic examination in all preterm gestations. We examined the intensity of FIR, and the frequency of FIRS, proven EONS, suspected EONS and chorioamnionitis according to the progression of inflammation in UC. Written informed consent was obtained from all patients. The Institutional Review Board of Seoul National University Hospital approved the collection and use of these samples and information for research purposes. The Seoul National University has a Federal Wide Assurance with the Office for Human Research Protections (OHRP) of the Department of Health and Human Services of the United States. The Institutional Review Board of Seoul National University Hospital specifically approved this study.

### FIR, FIRS and proven EONS, and suspected EONS

UC blood was collected in ethylene diaminetetraacetic acid (EDTA) containing blood collection tubes by venipuncture of UV at birth. Samples were then centrifuged and supernatants were stored in polypropylene tubes at -70°C. The intensity of FIR was gauged by umbilical cord plasma (UCP) CRP concentration (ng/ml) at birth. CRP concentrations in UCP were measured with a commercially available enzyme linked immunosorbent assay (Immunodiagnostik AG, Bensheim, Germany). The sensitivity of the test was 0.02 ng/ml. Both intra-and inter-assay coefficients of variation were <10%. FIRS was defined as an elevated UCP CRP concentration (≥ 200 ng/ml) at birth [7].

EONS was diagnosed in the presence of a positive blood culture result within 72 hours of delivery. EONS was suspected in the absence of a positive culture when two or more of the following criteria were present within 72 hours of delivery: 1) white blood cell count of < 5,000 cells/mm^3^; 2) polymorphonuclear leukocyte (PMN) count of < 1,800 cells/mm^3^; and 3) I/T ratio (ratio of bands to total neutrophils) > 0.2. These criteria have been previously used in the pediatric and obstetric literature [5, 8, 17].

### Chorioamnionitis, progression of inflammation in UC, and clinical chorioamnionitis

Placental tissue samples obtained for pathologic evaluation included chorioamniotic membrane roll (i.e., extraplacental membranes: CD and amnion), chorionic plate (CP), and UC. These samples were fixed in 10% neutral buffered formalin and embedded in paraffin. Sections of prepared tissue blocks were stained with hematoxylin and eosin (H & E). Pathologists were masked to the clinical information. Acute inflammation in extraplacental membranes was defined in the presence of neutrophil infiltration in either CD or amnion. Acute inflammation in CD, amnion and CP was diagnosed with the use of previously published criteria [18] as in the following; 1) choriodeciduitis was diagnosed in the presence of at least one focus of >5 neutrophils in the CD; 2) chorioamnionitis was diagnosed in the presence of at least one focus of > 5 neutrophils in the amnion; 3) CP inflammation was diagnosed in the presence of more than one focus of at least 10 neutrophilic collections or diffuse inflammation in the subchorionic fibrin, or diffuse and dense inflammation, neutrophilic infiltration into connective tissue of the chorionic plate, or chorionic vasculitis. Inflammation in UC was diagnosed in the presence of neutrophil infiltration into the umbilical vessel walls or WJ according to previously published criteria [18]. The progression of inflammation in UC was slightly modified from previously published criteria [19] and divided as in the following: 1) stage 0: inflammation-free UC; 2) stage 1: umbilical phlebitis only; 3) stage 2:: involvement of at least one UA and either the other UA or UV without extension into WJ; 4) stage 3: the extension of inflammation into WJ.

Clinical chorioamnionitis was diagnosed when maternal body temperature was elevated to 37.8°C and ≥ 2 of the following criteria were present according to the definitions previously described in detail [20]: uterine tenderness, malodorous vaginal discharge, maternal leukocytosis (> 15,000 cells/mm^3^), maternal tachycardia(>100 beats/min) and fetal tachycardia (>160 beats/min).

### Statistical analysis

We used Kruskal-Wallis test for the comparisons of continuous variables, and Pearson’s chi-square test for the comparisons of categorical variables according to the progression of inflammation in UC in clinical characteristics and pregnancy outcomes. Moreover, Fisher’s exact test with Bonferroni’s correction was used for the multiple comparisons of proportions between the groups according to the progression of inflammation in UC in clinical characteristics and pregnancy outcomes. Kruskal-Wallis test and Spearman rank correlation test were used to investigate the relationship between UCP CRP concentrations at birth and the progression of inflammation in UC. Pearson’s chi-square test was used to compare the frequency of FIRS, EONS and chorioamnionitis between groups according to the progression of inflammation in UC. We used the linear by linear association test for the assessment of trend. Multiple logistic regression analysis for the adjustment of confounding variables was used to explore the relationship of various independent variables including the progression of inflammation in UC with suspected EONS and chorioamnionitis. Statistical significance was defined as a p<0.05.

## Results

### Clinical characteristics and pregnancy outcomes of study population

We described clinical characteristics and pregnancy outcomes according to the progression of inflammation in UC in Table 1. Stage 0 (Inflammation-free UC), stage 1 (umbilical phlebitis only), stage 2 (involvement of at least one UA and either the other UA or UV without extension into WJ), and stage 3 (the extension of inflammation into WJ) of inflammation in UC were present in 48.1% (115/239), 15.5% (37/239), 6.7% (16/239), and 29.7% (73/239) of cases. There was a significant difference in the frequency of clinical chorioamnionitis and preterm-PROM as a cause of preterm delivery between the groups according to the progression of inflammation in UC (Table 1). However, there was no significant difference in other variables including GA at delivery between the groups (Table 1).

**Table 1:** Clinical characteristics and pregnancy outcomes according to the progression of inflammation in umbilical cord (UC).

	Inflammation-free UC	Inflammation in UC			
	Stage 0	Umbilical phlebitis only Stage 1	Involvement of at least one UA and either the other UA or UV without extension into WJ Stage 2	The extension of inflammation into WJ Stage 3 (n = 71)	P value ^a^
	(n = 115)	(n = 37)	(n = 16)		
	48.1% (115/239)	15.5% (37/239)	6.7% (16/239)	29.7% (71/239)	
Maternal age, year (mean ± SD)	29.0 ± 4.4	29.7 ± 5.1	29.8 ± 4.0	30.2 ± 4.0	NS
Nulliparity	51.3% (59/115)	45.9% (17/37)	37.5% (6/16)	43.7% (31/71)	NS
Clinical chorioamnionitis^†^	5.3% (6/114)	18.9% (7/37)	18.8% (3/16)	14.5% (10/69)	**0.045** ^b^
Preterm-PROM as a cause of preterm delivery	47.8% (55/115)	62.2% (23/37)	75.0% (12/16)	67.6% (48/71)	**0.021** ^c^
Male Newborn	58.3% (67/115)	59.5% (22/37)	56.2% (9/16)	46.5% (33/71)	NS
Cesarean delivery	30.4% (35/115)	32.4% (12/37)	31.2% (5/16)	28.2% (20/71)	NS
Median GA at delivery, wks [range]	32.9 [21.6–36.0]	32.1 [23.3–35.7]	31.9 [25.4–35.9]	31.3 [21.9–36.0]	NS
Birth weight, g (mean ± SD)	1798 ± 691	1766 ± 642	1734 ± 567	1659 ± 618	NS
1 min Apgar score of < 7	49.6% (57/115)	56.8% (21/37)	43.8% (7/16)	60.6% (43/71)	NS
5 min Apgar score of < 7	33.9% (39/115)	37.8% (14/37)	12.5% (2/16)	35.2% (25/71)	NS

*GA*, gestational age; *NS*, not significant; *preterm-PROM*: preterm premature rupture of membranes; *SD*, standard deviation; *UA*, umbilical artery; *UC*, umbilical cord; *UV*, umbilical vein; *WJ*, Wharton’s jelly.

^a^, Intergroup difference by Pearson’s chi-square test (categorical variables) and Kruskal-Wallis test (continuous variables)

^b^, There was no difference in clinical chorioamnionitis between any of the groups by Fisher’s exact test with Bonferroni’s correction.

^c^, There was no difference in preterm-PROM as a cause of preterm delivery between any of the groups by Fisher’s exact test with Bonferroni’s correction.

^†^ Of 239 cases, 236 patients were included in this analysis, because the information about clinical chorioamnionitis in medical record was omitted in 3 patients.

### UCP CRP concentrations at birth according to the progression of inflammation in UC

**Fig 1:**
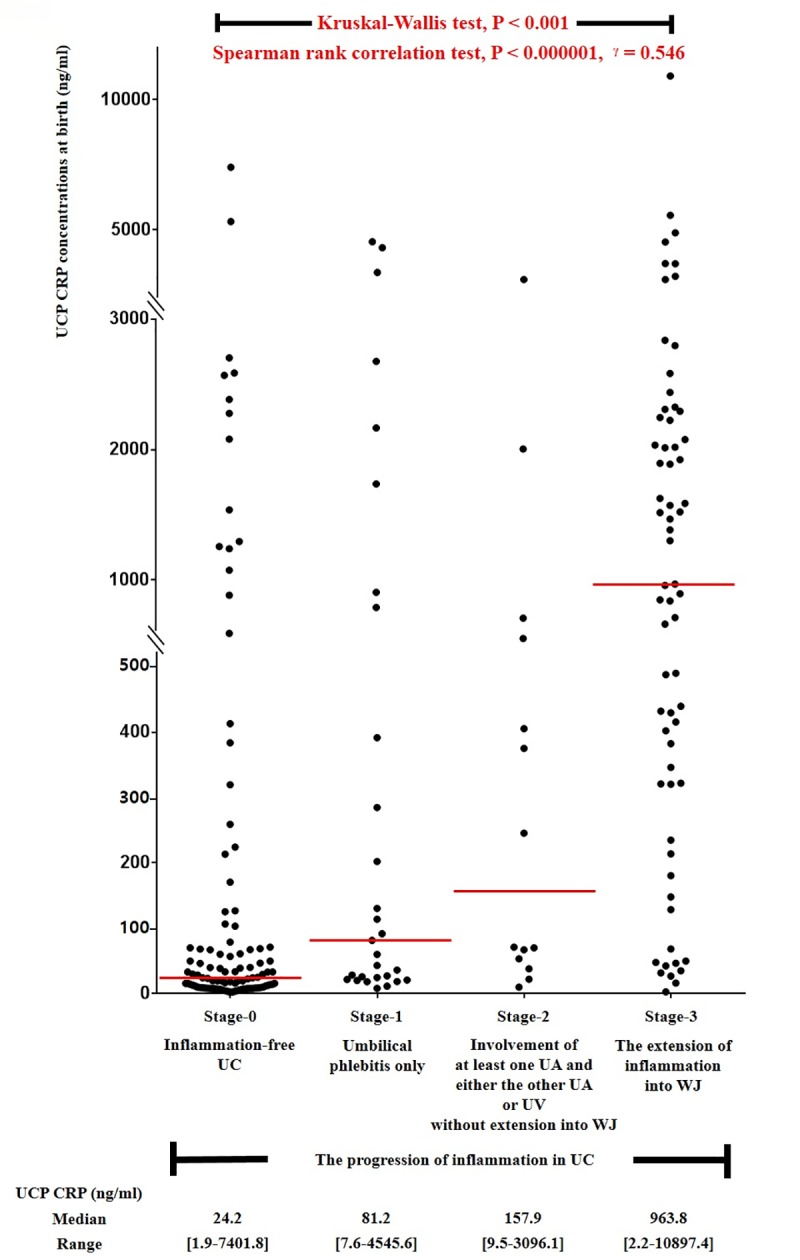
Relationship between the progression of inflammation in umbilical cord (UC) and umbilical cord plasma (UCP) CRP concentrations at birth (ng/ml). Kruskal-Wallis test and Spearman rank correlation test were performed, and each P value is shown in graph. The median value and range of UCP CRP concentrations at birth (ng/ml) according to the progression of inflammation in UC are also shown in graph. Of 239 cases which met the entry for this study, 215 patients had UCP CRP concentrations at birth; however, 24 patients did not have an UCP CRP concentration at birth because of the limited amount of the remaining UCP.

Fig 1 shows the relationship between the progression of inflammation in UC and UCP CRP concentrations at birth (ng/ml). UCP CRP concentrations at birth as an intensity of FIR continuously increased according to the progression of inflammation in UC (Fig 1: Kruskal-Wallis test, P < 0.001; Spearman rank correlation test, P <0.000001, r = 0.546).

### FIRS, proven EONS, suspected EONS and chorioamnionitis according to the progression of inflammation in UC

**Fig 2:**
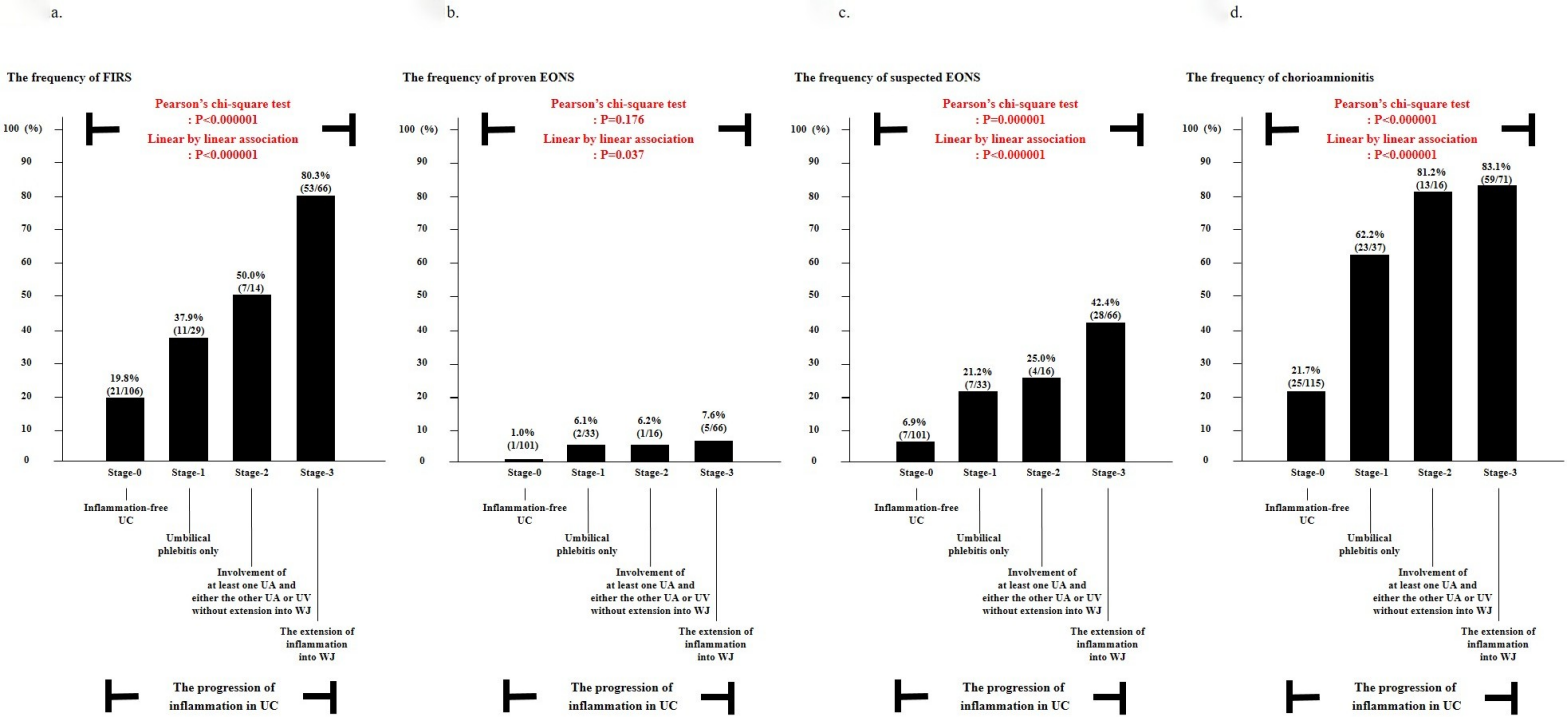
**The frequency of fetal inflammatory response syndrome (FIRS) [a], proven early-onset neonatal sepsis (EONS) [b], suspected EONS [c] and chorioamnionitis [d] according to the progression of inflammation in UC.** Each P value is shown in graph. Of 239 cases which met the entry for this study, 215 patients had UCP CRP concentrations at birth; however, 24 patients did not have an UCP CRP concentration at birth because of the limited amount of the remaining UCP, and therefore, we could not examine the frequency of FIRS. Moreover, twenty-three neonates were excluded from the analysis in the evaluation of proven EONS and suspected EONS because they died shortly after delivery as a result of extremely prematurity (n = 19) or anomaly (n = 4) and thus could not be evaluated with respect to the presence or absence of proven EONS and suspected EONS.

Fig 2 illustrates the frequency of FIRS (Fig 2A), proven EONS (Fig 2B), suspected EONS (Fig 2C), and chorioamnionitis (Fig 2D) according to the progression of inflammation in UC. There was a significant and stepwise increase in FIRS, suspected EONS and chorioamnionitis according to the progression of inflammation in UC (each for P <0.000005 in both Pearson’s chi-square test and linear by linear association). Multiple logistic regression analysis demonstrated that the more advanced stage in the progression of inflammation in UC (i.e., stage 1 vs. stage 2 vs. stage 3), the better predictor of suspected EONS (Table 2) (Odds ratio [OR] 3.358, 95% confidence interval [CI] 1.020–11.057 vs. OR 5.147, 95% CI 1.189–22.275 vs. OR 11.040, 95% CI 4.118–29.592) and chorioamnionitis (Table 3) (OR 6.593, 95% CI 2.717–15.999 vs. OR 16.508, 95% CI 3.916–69.596 vs. OR 20.167, 95% CI 8.629–47.137) even after the correction of potential confounding variables such as GA at delivery.

**Table 2:** Relationship of various independent variables with suspected early-onset neonatal sepsis (EONS) by overall logistic regression analysis.

	Odds ratio	95% CI	*P value*
Stage 1^†^ of inflammation in UC	3.358	1.020–11.057	0.046
Stage 2^‡^ of inflammation in UC	5.147	1.189–22.275	0.028
Stage 3^∫^ of inflammation in UC	11.040	4.118–29.592	0.000002
GA at delivery	1.138	0.848–1.527	NS
Preterm-PROM as a cause of spontaneous preterm delivery	0.756	0.340–1.681	NS
Clinical chorioamnionitis	3.154	1.091–9.117	0.034
Cesarean section	1.230	0.555–2.728	NS
Birth weight	1.000	0.998–1.001	NS
5 min Apgar score < 7	2.749	1.015–7.447	0.047

^†^Umbilical phlebitis only

^‡^Involvement of at least one UA and either the other UA or UV without extension into WJ

^∫^The extension of inflammation into WJ

*CI*, confidence interval; *GA*, gestational age; *NS*, not significant; *preterm-PROM*, preterm premature rupture of membranes; *UA*, umbilical artery; *UV*, umbilical vein; *WJ*, Wharton’s jelly

**Table 3:** Relationship of various independent variables with chorioamnionitis by overall logistic regression analysis.

	Odds ratio	95% CI	*P value*
Stage 1^†^ of inflammation in UC	6.593	2.717–15.999	0.00003
Stage 2^‡^ of inflammation in UC	16.508	3.916–69.596	0.000134
Stage 3^∫^ of inflammation in UC	20.167	8.629–47.137	< 0.000001
GA at delivery	0.990	0.791–1.239	NS
Preterm-PROM as a cause of spontaneous preterm delivery	1.329	0.657–2.687	NS
Clinical chorioamnionitis	1.882	0.586–6.044	NS
Cesarean section	0.722	0.350–1.491	NS
Birth weight	0.999	0.998–1.000	NS
5 min Apgar score < 7	0.835	0.316–2.209	NS

^†^Umbilical phlebitis only

^‡^Involvement of at least one UA and either the other UA or UV without extension into WJ

^∫^The extension of inflammation into WJ

*CI*, confidence interval; *GA*, gestational age; *NS*, not significant; *preterm-PROM*, preterm premature rupture of membranes; *UA*, umbilical artery; *UV*, umbilical vein; *WJ*, Wharton’s jelly

## Discussion

### Principal finding of this study

Principal finding of this study is that the intensity of FIR, and the frequency of FIRS, EONS and chorioamnionitis are continuously increased according to the progression of inflammation in UC in cases with both inflammation in extraplacental membranes or CP and preterm delivery due to either PTL or preterm-PROM.

### FIR and FIRS according to the progression of inflammation in UC

We firstly demonstrated that the intensity of FIR (Fig 1) and the frequency of FIRS (Fig 2A) continuously increase according to the progression of inflammation in UC. Of note, the progression of inflammation in UC in our study (i.e., stage 0, inflammation-free UC; stage 1, umbilical phlebitis only; stage 2, involvement of at least one UA and either the other UA or UV without extension into WJ; stage 3, the extension of inflammation into WJ) was slightly modified from the previously reported criteria [19] based on the widely accepted pathophysiology of the migration of fetal neutrophil in UC [9]. However, previous studies examining the intensity of FIR according to the progression of inflammation in UC [10, 12, 13] had the limitation in either the grouping of inflammation in UC [12, 13] (Table 4) or the number of study population [10, 13] as in the following: 1) Kim CJ et al. demonstrated that cases with inflammation in UC but without umbilical arteritis (n = 49) had higher UCP IL-6 concentrations at birth than those without inflammation in UC (n = 528), but lower UCP IL-6 concentrations at birth than those with umbilical arteritis (n = 59) irrespective of the status of the extension of neutrophils into WJ in 636 cases of preterm delivery (GA at delivery < 36 weeks) [12]. However, UCP IL-6 concentration at birth were not compared between the extension of neutrophils into WJ beyond umbilical vessels and either inflammation in UC but without umbilical arteritis or umbilical arteritis although a large number of cases were included in that study [12]; 2) Salafia CM et al. reported that the presence of extension of neutrophils into WJ was associated with a more increased FIR gauged by IL-1, IL-2R and IL-6 than the absence of that lesion although being examined in a small number of preterm gestation (n = 32) due to PTL [10]. However, this increasing pattern in FIR disappeared according to the sequential progression of inflammation in UC (i.e., inflammation-free UC, inflammation restricted to UV, inflammation in at least one UA and either the other UA or UV without extension into WJ, and the extension of inflammation into perivascular WJ, and the extension of inflammation into deep WJ) [10]; 3) Rogers BB et al. showed that cases with necrotizing funisitis did not have higher umbilical venous IL-6 concentrations at delivery than those with inflammation restricted to three vessels in 110 cases of preterm gestation [13]. Moreover, that study did not consider the presence of inflammation in WJ for staging of inflammation in UC, and did not differentiate between the involvement of UV and UA in inflammation restricted to umbilical vessels [13].

**Table 4:** Classification of the progression of inflammation in umbilical cord in various studies.

Study [Reference]	Stages, grades or groups of inflammation in umbilical cord	Inclusion of necrotizing funisitis as an independent stage	Differentiation in inflammation of umbilical vessels between UV and UA	Inclusion of inflammation in WJ as an independent stage	Differentiation between inflammation restricted to umbilical vessels and inflammation of WJ	Marker as a FIR
Rogers BB et al., 2002 [13]	Group 1: one vessel with vasculitis Group 2: two vessels with vasculitis Group 3: three vessels with vasculitis Group 4: necrotizing funisitis (the presence of necrotic debris surrounding or involving the umbilical vessels)	Yes	No	No	No	IL-6
van Hoeven KH et al., 1996 [21]	Stage 1: Acute funisitis limited to the vein alone Stage 2: Acute funisitis involving one or more arteries	No	Yes	No	No	N/A
Kim CJ et al., 2001 [12]	Group 1: inflammatory lesions limited to the vein with or without inflammation of the WJ Group 2: umbilical arteritis with or without inflammation of the WJ or phlebitis	No	Yes	No	No	IL-6
Redline RW et al., 2003 [22]	FIR stage 1: Umbilical phlebitis, and extension of neutrophils into WJ is allowed if not aggregated in a concentric band, ring or halo around UV FIR stage 2: Umbilical vasculitis (one or two arteritis ± vein) or umbilical panvasculitis (all vessels), and extension of neutrophils into WJ is allowed if not aggregated in a concentric band, ring or halo around umbilical vessel(s). FIR stage 3: Necrotizing funisitis or concentric umbilical perivasculitis (PMN, cellular debris, eosinophilic precipitate, and/or mineralization in a concentric band, ring or halo around one or more umbilical vessels)	Yes	Yes	No	No	N/A
Blanc WA, 1981 [23]	Stage I: Margination of PMNs against the endothelium of umbilical vessels Stage II: Umbilical vascular wall invasion of PMNs Stage III: Spread of PMNs in WJ	.No	No	Yes	Yes	N/A
Wharton KN et al., 2004 [24]	Grade 1: one vessel vasculitis Grade 2: two vessel vasculitis Grade 3: PMNs in WJ with one or two vessel vasculitis Grade 4: PMNs in WJ with vasculitis of all three vessels	No	No	Yes	Yes	N/A
Dexter SC et al., 2000 [25]	Grade 1: one vessel with vasculitis (PMNs in vessel wall) without PMNs in WJ Grade 2: two- or three-vessel vasculitis without PMNs in WJ Grade 3: PMNs in WJ regardless of the presence of vasculitis	No	No	Yes	Yes	N/A
Yoon BH et al., 1995 [18]	Grade 1: Neutrophilic infiltration confined to umbilical vessel walls Grade 2: Extension of neutrophilic infiltration into WJ	No	No	Yes	Yes	IL-6
Salafia CM et al., 1989 [19] Salafia CM et al., 1997 [10]	Grade 1: PMNs within the inner third of the UV wall Grade 2: PMNs within the inner third of at least two umbilical vessel walls (including at least one UA wall) Grade 3: PMNs in the perivascular WJ Grade 4: Panvasculitis and funisitis extending deep into WJ	No	Yes	Yes	Yes	IL-1β, IL-2R, Il-6

*FIR*, fetal inflammatory response; *N/A*, not available; *PMN*, polymorphonuclear leukocytes; *UA*, umbilical artery; *UC*, umbilical cord; *UV*, umbilical vein; *WJ*, Wharton’s jelly.

### EONS according to the progression of inflammation in UC

An increased FIR is known to be associated with EONS [6–8]. Therefore, considering that the intensity of FIR continuously increased according to the progression of inflammation in UC in our study, one might expect that the progression of inflammation in UC is related to a more frequent EONS. Indeed, the frequency of EONS was increased with the sequential progression of inflammation in UC (Fig 2B and 2C). We could find only one study examining the relationship between the progression of inflammation in UC and EONS, which demonstrated that there was no significant intergroup difference in the frequency of EONS between each stage of acute histologic funisitis and no significant trend in the frequency of EONS according to the progression of stage in acute histologic funisitis among 339 cases of early preterm delivery due to preterm-PROM [14]. However, in that study, the involvement of inflammation in WJ, which is critical for FIR [10], was not used for the differentiation of stage in acute histologic funisitis [14]. Therefore, no relationship between the stage of acute histologic funisitis and EONS might be attributed to this classification itself.

### Chorioamnionitis according to the progression of inflammation in UC

Chorioamnionitis develops just before the occurrence of inflammation in UC which is known to be the final stage of AIUI [1–3]. Moreover, chorioamnionitis is considered to be a more advanced stage than choriodeciduitis only in the context of inflammation in extraplacental membranes, that is the MIR of placenta [4, 5]. Therefore, it is plausible that chorioamnionitis is more frequent according to the progression of inflammation in UC. However, up to now, there is no information about the relationship between the progression of inflammation in UC and chorioamnionitis. We firstly reported that chorioamnionitis, as an advanced stage of MIR in placenta, continuously increased according to the progression of inflammation in UC (Fig 2D). This is consistent with the observation of our previous report, which demonstrated that the involvement of amnion in AIUI is an indicator that a FIR is more likely and severe [5].

### Staging of inflammation in UC

Table 4 shows the various classifications of the progression of inflammation in UC used in previous studies [10, 12, 13, 18, 19, 21–25], which means that no classification is unanimously accepted. Some of classifications did not use the involvement of inflammation into WJ for staging of inflammation in UC [12, 13, 21, 22], while others did not differentiate between inflammation restricted to UV and inflammation in UA in the context of inflammation restricted to the umbilical vessels although they used the involvement of inflammation into WJ for the differentiation of inflammation in UC [16, 23–25]. However, it is sensible for us to differentiate between inflammation restricted to UV and inflammation in UA in cases without inflammation into WJ, and between inflammation restricted to umbilical vessels and inflammation into WJ for the classification of the progression of inflammation in UC, in the light of the evidence that a FIR is significantly higher in cases with umbilical arteritis than in those with umbilical phlebitis only irrespective of the status of inflammation in WJ [12] and in cases with inflammation into WJ than in those with inflammation restricted to umbilical vessels [10]. Therefore, we slightly modified the criteria of the progression of inflammation in UC by Salafia CM et al. [19] as in the following: 1) stage 1: umbilical phlebitis only; 2) stage 2: involvement of at least one UA and either the other UA or UV without extension into WJ; 3) stage 3: the extension of inflammation into WJ. Indeed, we firstly demonstrated that the intensity of FIR and the frequency of FIRS, EONS and chorioamnionitis were continuously increased according to the progression of inflammation in UC with the use of this new classification (Figs 1 and 2).

### Strengths and weakness of the study

Major strengths of the study are as in the followings. Firstly, our study had a large cohort (n = 239) of preterm delivery (≤36weeks) with inflammation in extraplacental membranes or CP. Therefore, the frequency of inflammation in UC (52%) in our study was much higher than that in other previous preterm studies (i.e., 17% in Kim’s study [12] and 17% in Rogers’ study [13]) due to the inclusion criteria of our study, and moreover, cases with isolated inflammation in UC, which was generally defined in the presence of inflammation in UC in patients without inflammation in other placental compartments (i.e., CD, amnion and CP), were not included in this study. In general, inflammation in UC develops after the occurrence of acute-HCA in either extraplacental membranes or CP [1–3]. However, there is always the possibility of cases with isolated inflammation in UC in the absence of inflammation in extraplacental membranes or CP although such cases are relatively infrequent. The development of isolated inflammation in UC is inconsistent with the general progression of AIUI [1–3]. Therefore, fetal inflammatory milieu may be different between ‘isolated inflammation in UC’ and ‘inflammation in UC with inflammation in extraplacental membranes or CP’, and this difference of ‘isolated inflammation in UC’ from ‘inflammation in UC with inflammation in extraplacental membranes or CP’ may lead to the distorted findings about the relationship between the progression of inflammation in UC and either FIR or EONS during AIUI. Secondly, our study did not have cases of preterm delivery due to maternal fetal indication (i.e., preeclampsia and fetal growth restriction [FGR]). Therefore, we could examine FIR, EONS and chorioamnionitis according to the progression of inflammation in UC in cases with preterm birth due to only spontaneous causes (i.e., either PTL or preterm-PROM), which is closely associated with AIUI. Of note, recent animal studies demonstrated that chronic hypoxemia with absent bacterial infection resulted in mildly growth restricted offspring and increased IL-6 and TNF-α in fetal sera [26, 27]. Moreover, FGR was associated with an elevated FIR in some human studies [28–30]. Therefore, FGR may be associated with an increased FIR irrespective of AIUI and subsequent inflammation in UC, leading to the skewed results about the relationship between the progression of inflammation in UC and FIR. The weaknesses of our study are as in the following: 1) We could not examine the pathophysiologic significance of isolated inflammation in UC (that is, unexplained FIR) related to meconium because this pathologic entity was not included in our current study. Previous study in our institution demonstrated that 8.3% of term neonates with meconium aspiration syndrome had isolated inflammation in UC [31], and therefore, there is a good chance meconium is associated with FIR via an independent pathophysiologic mechanism (i.e., direct and chemical stimulation of meconium on UC leading to inflammation in UC) from traditional pathways of AIUI. However, the analysis of either unexplained FIR or isolated inflammation in UC related to meconium is beyond the scope of our current study; 2) We have obtained a random section from UC, according to the method previously described by Kim CJ et al. [32]. Of note, Katzman PJ et al. demonstrated that acute inflammation developed the proximal section of UC in twenty-one cases (95%) and the distal section in only one case (5%) of 22 cases with inflammation in one or both arteries involved in one cord section only (P <0.0001), while involvement of inflammation was divided almost equally between the proximal (n = 41) and distal (n = 42) sections of UC among 83 cases with umbilical phlebitis in only one section of UC through the pathologic examination of two sections (i.e., proximal and distal sections) in UC, leading to the proposal that at least 2 cord sections representing proximal and distal sites are recommended to exclude FIR [33]. This unique difference in the distribution of inflammation of proximal and distal sections between UV and UA may be explained by the difference in blood flow velocity between both UV and UV [33]. Venous blood flow is relatively slower than flow in the arteries through most of cord length, and this slower velocity may allow neutrophils to marginate anywhere in UV along the cord length [33]. On the contrary, arterial blood is under higher pressures through most of UC, which results in a higher velocity [33]. However, umbilical arterial blood slows down or arterial flow decreases at an only proximal section within 5 cm of the cord insertion site into the placental fetal surface, as it enters the watershed of the chorionic vessel network, where high-capacitance/low-resistance blood is distributed to the chorionic villi [33– 35]. This blood flow deceleration within this segment of UC is due to the Hyrtl anastomosis, which joins the 2 arteries just before they enter CP, effectively equalizing the pressures between the 2 arteries and contributing to a decreased end diastolic flow [33–35]. This decrease in velocity should allow for more effective neutrophil margination from UA in the proximal portion of UC [33]. However, at present, there are no agreed standards for placental sampling or storage of samples for placental evaluation [36]. Indeed, most previous publications reporting the frequency of inflammation in UC performed only one section of UC [14, 21, 22, 37–43]

### Clinical implications

We demonstrated that there is a stepwise increase in FIRS, EONS and chorioamnionitis according to the progression of inflammation in UC. Therefore, the major implication of our study is that we can propose that there is a better chance for the development of EONS when inflammation in UC is present in WJ (stage 3) than when either it is restricted to UV only (stage 1) or it develops in UA without extension into WJ (stage 2) (Table 2). This information will help neonatologists to predict the development of EONS and treat EONS prophylactically in preterm newborn with inflammation in UC but without the information of FIR at birth in spite of the heterogeneity of inflammation in UC. Moreover, our findings may suggest that funisitis (inflammation in UC) is both qualitatively and quantitatively histologic counterpart of FIRS (Fig 2A), and a surrogate marker for an advanced stage of MIR, chorioamnionitis (Fig 2D and Table 3).

### Unanswered questions and proposals for future study

To the best of our knowledge, this is the first report that the sequential progression of inflammation rather than its mere presence in UC is more important in both FIR and EONS. However, up to now, it is not yet known how FIR develops through interaction between chorionic vasculitis and the progression of inflammation in UC, although FIR is attributed to chorionic vasculitis in addition to inflammation in UC. Ultimately, we should explore the relationship among chorionic vasculitis, the progression of inflammation in UC, and FIR.

## Supporting information

S1 Appendix Umbilical cord plasma CRP concentrations in stages according to the progression of inflammation in umbilical cord. (XLSX)

## Abbreviation

AIUI: ascending intrauterine infection
CD: choriodecidua
CP: chorionic plate
CRP: C-reactive proteins
EDTA: ethylene diaminetetraacetic acid
EONS: early-onset neonatal sepsis
FGR: fetal growth restriction
FIR: fetal inflammatory response
FIRS: fetal inflammatory response syndrome
GA: gestational age
H & E: hematoxylin and eosin
MIR: maternal inflammatory response
PMN: polymorphonuclear leukocyte
Preterm-PROM: preterm premature rupture of membranes
PTL: preterm labor and intact membranes
ROM: rupture of membranes
UA: umbilical artery
UAs: umbilical arteries
UC: umbilical cord
UCP: umbilical cord plasma
UV: umbilical vein
WJ: Wharton’s jelly
